# A Pilot Study on Peptidylarginine Deiminases and Protein Deimination in Animal Cancers across Vertebrate Species

**DOI:** 10.3390/ijms23158697

**Published:** 2022-08-04

**Authors:** Jameel M. Inal, Mariya Hristova, Sigrun Lange

**Affiliations:** 1 School of Life and Medical Sciences, University of Hertfordshire, Hatfield AL10 9AB, UK; 2 School of Human Sciences, London Metropolitan University, London N7 8DB, UK; 3 Perinatal Brain Repair Group, Department of Neonatology, UCL Institute for Women’s Health, London WC1E 6HU, UK; 4 Tissue Architecture and Regeneration Research Group, School of Life Sciences, University of Westminster, London W1W 6UW, UK

**Keywords:** peptidylarginine deiminase (PAD), deimination/citrullination, deiminated histone H3 (CitH3), cancer, cancer evolution, phylogeny

## Abstract

PADs are a group of calcium-dependent enzymes that play key roles in inflammatory pathologies and have diverse roles in cancers. PADs cause irreversible post-translational modification of arginine to citrulline, leading to changes in protein function in different cellular compartments. PAD isozyme diversity differs throughout phylogeny in chordates, with five PAD isozymes in mammals, three in birds, and one in fish. While the roles for PADs in various human cancers are mounting (both in regards to cancer progression and epigenetic regulation), investigations into animal cancers are scarce. The current pilot-study therefore aimed at assessing PAD isozymes in a range of animal cancers across the phylogeny tree. In addition, the tissue samples were assessed for total protein deimination and histone H3 deimination (CitH3), which is strongly associated with human cancers and also indicative of gene regulatory changes and neutrophil extracellular trap formation (NETosis). Cancers were selected from a range of vertebrate species: horse, cow, reindeer, sheep, pig, dog, cat, rabbit, mink, hamster, parrot, and duck. The cancers chosen included lymphoma, kidney, lung, testicular, neuroendocrine, anaplastic, papilloma, and granulosa cell tumour. Immunohistochemical analysis revealed that CitH3 was strongly detected in all of the cancers assessed, while pan-deimination detection was overall low. Both PAD2 and PAD3 were the most predominantly expressed PADs across all of the cancers assessed, while PAD1, PAD4, and PAD6 were overall expressed at lower, albeit varying, levels. The findings from this pilot study provide novel insights into PAD-mediated roles in different cancers across a range of vertebrate species and may aid in the understanding of cancer heterogeneity and cancer evolution.

## 1. Introduction

PADs are a group of calcium-dependent enzymes which play key roles in a number of inflammatory and autoimmune pathologies and have diverse roles in cancers, including via epigenetic regulation and modulation of proteins involved in cancer progression [1,2,3]. While five PAD isozymes are present in mammals (PAD1, 2, 3, 4, and 6), PAD isozyme diversity differs throughout phylogeny in chordates, with three PAD isoforms in birds and reptiles but only one PAD form (PAD2-like) in fish [4,5,6]. In addition, PAD homologues (arginine deiminases, ADI) are found in bacteria, fungi, and parasites, and PADs seem to have emerged in Chordata via horizontal gene transfer (possibly from cyanobacteria) [7,8].

PADs cause an irreversible post-translational protein modification by the conversion of arginine to citrulline, which can lead to changes in protein folding and function, contribute to neo-epitope formation, and affect proteins in different cellular compartments, including the cytoplasm, mitochondria, and the nucleus [9]. PAD isozymes display some differences in their tissue-specific distribution, some of which overlap, and PAD2 is considered the most ancestral and ubiquitously expressed PAD isozyme [1,3,4]. Activation of PADs is catalysed by calcium and PAD-mediated downstream citrullination/deimination is closely related to a number of pathologies, while PADs also have various physiological roles. PAD1 has been strongly associated with skin physiology and skin diseases [10], as well as with embryo development [11,12], while novel roles in breast cancer metastasis and epithelial-to-mesenchymal transition (EMT) have been identified [13]. PAD3 is associated with skin physiology but also with central nervous system (CNS) regeneration and neuronal cell stem-ness [14,15,16,17], as well as more recently with emerging critical roles in cancers, including in aggressive cancers [18,19,20]. PAD2 and PAD4 have been strongly associated to both autoimmune diseases [21] and cancers [1,22]. Importantly, while research on PAD4 has dominated in relation to cancer for a considerable time, roles for the other PAD isozymes are increasingly emerging, including for PAD2, PAD3, and PAD1. PAD6 is mainly associated with developmental processes, including early embryo development and pre-implantation [23,24,25,26,27,28], but roles in cancers remain to be investigated. Interestingly, while some PAD isoforms have been found to be overexpressed in a range of human cancers, including increased PAD2 expression in blood of patients [29], downregulation of PADs has also been observed in some cancers [30]. The PADs are cytoplasmic enzymes with the ability to translocate to the nucleus, and while PAD4 is the only isozyme that contains a classic nuclear translocation site, other PAD forms have also been localised to the nucleus, including PAD2 and PAD3. Furthermore, PAD1, PAD2, 3, and 4 have all been found to be involved in gene regulation via deimination of histones [11,16,17,31].

Overall, evidence for the diverse roles of PADs and deimination in human cancers is mounting both regarding cancer progression and epigenetic regulation [32], while investigations into animal cancers remain scarce. To date, two main studies have been published on animal cancers (excluding animal cancer models such as murine, for human disease), assessing PAD2 expression in canine, feline, and human mammary tumours, including in relation to histone citrullination [31,33]. Furthermore, the use of pharmacological pan-PAD inhibition has been explored in canine and feline mammary cancer cell lines and associated xenograft models [34]. Roles for other PAD isoforms remain to be investigated, including also in a wider array of naturally occurring animal cancers.

Increased understanding of PAD isozyme expression and protein deimination in animal cancers may be of considerable importance as varying roles for the different PAD isozymes in human cancer types are increasingly being identified. The current study was therefore aimed at carrying out a pilot-screen of PAD isoform expression and designed to detect protein deimination in a range of cancers across different vertebrate species including birds (parrot and duck), rabbit, hamster, cat, dog, mink, sheep, pig, reindeer, cow, and horse. Our findings provide some novel insights into PAD expression in a range of animal cancers and may aid understanding both of PAD-mediated roles in different cancers as well as provide insights into possible PAD-mediated roles in cancer evolution.

## 2. Results

### 2.1. Detection of Protein Citrullination/Deimination, Deiminated Histone H3 and PAD Isoforms in Animal Cancer Tissues

**Figure 1:**
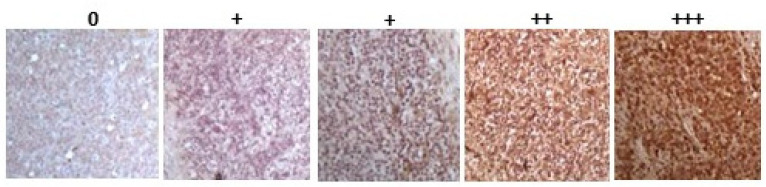
Scoring range/index (0, +, ++, +++) for immunohistochemical detection intensity of PADs, total deiminated proteins (F95) and histone H3 deimination (CitH3), as indicated for the different samples in Table 1.

Histological tissue sections were assessed for immunohistochemistry staining of PAD1, PAD2, PAD3, PAD4, and PAD6; for pan-citrullination/deimination using the F95 pan-deimination antibody; and for deiminated histone H3 using the anti-histone H3 (citrulline R2 + R8 + R17; CitH3) antibody. Scoring was performed according to staining intensity determined on a scale of 0–3, indicated by crosses as: + to +++; with brackets indicating lower expression than a full cross (see key in Figure 1). Table 1 summarises the results for immunohistochemical detection of PAD isozymes, CitH3, and F95 in all of the animal cancer tissue specimens assessed in the current study.

**Table 1:** Scoring for immunohistochemical staining results of the different histological tumour samples assessed in the current study. Staining intensity for each antibody, in each tumour sample, is based on the representative scoring index shown in Figure 1. Immunohistochemical staining results for each tumour sample assessed are shown for: pan-deimination staining using the F95 antibody, deiminated histone H3 (CitH3), and PAD isozymes 1–6. Animal species, tumour type, and staining intensity are listed.

Species	Tumour	F95	CitH3	PAD1	PAD2	PAD3	PAD4	PAD6
Rabbit *(Oryctolagus cuniculus)*	Mammary adenocarcinoma	+	++	++	+++	+++	++	++
Dog *(Canis lupus familiaris)*	(1) Mixed germ cell-sex cord stromal tumour, testicle. (2) Squamous cell tumour; origin of tissue unknown	(+)	++	+	+++	++	++	+
Regent parrot *(Polytelis anthopeplus)*	Lymphoma, liver and spleen	+	+++	+	++	+++	+	+
Domestic duck *(Anas platyrhynchos domesticus)*	Malignant round cell tumour, liver; probably lymphoma	+	+++	++	+++	+++	+	+
Mink (*Neovision vision)*	Malignant renal tumour, possibly nephroblastoma. Tumour in kidney with metastasis to lung and liver	(+)	+++	(+)	+++	++	+	++
Mink *(Neovision vision)*	Malignant renal tumour, probably nephroblastoma. Extensive necrosis and haemorrhage in tumour mass.	+	+++	++	+++	++	+	+
Cat *(Felis catus)*	Pulmonary carcinoma	+	+++	++	+++	+++	++	+
Horse *(Equus caballus)*	Carcinoma in liver (ovary and mesenteric lymph node)	(+)	(+)	0	+++	+++	+	++
Cattle *(Bos Taurus)*	Neuroendocrine tumour (metastasis to the heart)	(+)	+++	++	+++	+++	+	++
* Dog *(Canis lupus familiaris)*	* Anaplastic mammary carcinoma	++	+++	++	+++	++	++	++
Sheep *(Ovis aries)*	Malignant round cell tumour, skin	(+)	+++	(+)	+++	+	(+)	(+)
Hamster *(Mesocricetus auratus)*	Lymphoma, mesentery/small intestine	(+)	+	++	+++	+++	+	(+)
Dog *(Canis lupus familiaris)*	Scirrhous adenocarcinoma, mammary gland	+	+++	++	+++	++	+	+
Horse *(Equus caballus)*	Penile squamous cell carcinoma	0	+++	(+)	+++	+++	++	+
Cattle *(Bos Taurus)*	Metastatic carcinoma, lungs and lymph nodes	0	+++	+	+++	++	++	+
Cattle *(Bos Taurus)*	Metastatic carcinoma, lungs and lymph nodes	(+)	+++	++	+++	+++	++	+
Cat *(Felis catus)*	Malignant tumour, kidneys; prob. Lymphoma	+	+++	+	+++	+++	+	+
Dog *Canis lupus familiaris*	Granulosa cell tumour, ovary	+	+++	++	+++	++	+	+
Cattle *(Bos Taurus)*	Lymphoma, lymph node	0	+++	++	+++	+++	0	0
Reindeer *(Rangifer tarandus)*	Multicentric malignant tumour, probably mesothelioma	+	+++	++	+++	++(+)	++	++
Pig *(Sus scrofa domesticus)*	Lymphoma (lymph node)	(+)	+++	(+)	+++	+++	+	+

* Poor quality sections and therefore not included for imaging.

Representative images of the histological sections of the tissues analysed are presented in Figure 2, Figure 3, Figure 4, Figure 5, Figure 6, Figure 7, Figure 8, Figure 9, Figure 10, Figure 11, Figure 12, Figure 13, Figure 14, Figure 15, Figure 16, Figure 17, Figure 18, Figure 19, Figure 20 and Figure 21, showing immunohistochemical detection of PAD isozymes, pan-deimination (F95) and histone H3 citrullination/deimination (CitH3). Negative control images, showing secondary antibody only controls, are provided in Supplementary Figure S1.

**Figure 2:**
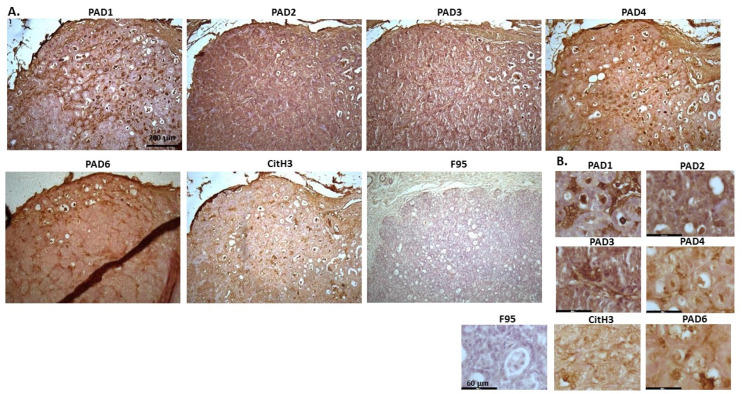
Rabbit, mammary adenocarcinoma. Biopsy from mammary gland of a four-year old female rabbit. (**A**) Overview images for PAD1, 2, 3, 4, 6, CitH3 and F95 stainings of serial tissue sections; scale bar represents 200 μm for all images; 10× objective. (**B**) Higher magnification shown for each staining from the images in (**A**); scale bar represents 60 μm for all images; 40× objective.

Figure 2 shows rabbit mammary adenocarcinoma; a biopsy from the mammary gland of a four-year-old female rabbit. The highest detection of PADs was seen for PAD2 and PAD3, while the other PAD isozymes were also detected. CitH3 was clearly positive, while pan-deimination was overall low. Staining for PAD1 was cytoplasmic as well as some strong nuclear stain. PAD2 and PAD3 showed diffuse cytoplasmic stain; PAD4 showed some nuclear staining, also with some cytoplasmic detection, while PAD6 showed more diffuse staining which was mainly cytoplasmic. CitH3 showed some nuclear staining and F95 positive staining was faint cytoplasmic.

**Figure 3:**
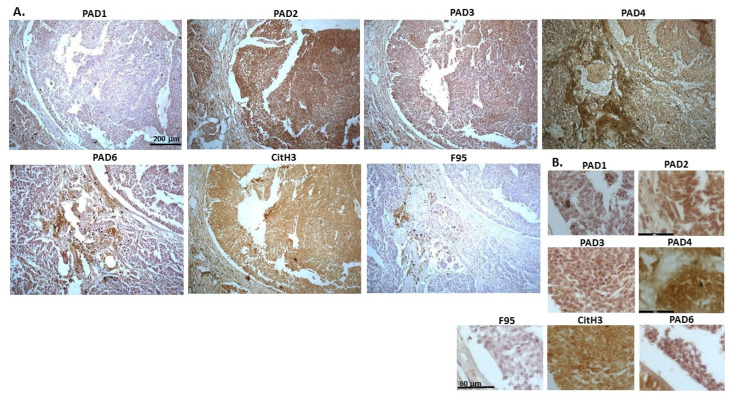
Dog, mixed germ cell-sex cord stromal tumour, testicle. Biopsies from a seven-year old dog, male. (**A**) Overview images for PAD1, 2, 3, 4, 6, CitH3 and F95 stainings of serial tissue sections; scale bar represents 200 μm for all images; 10× objective. (**B**) Higher magnification shown for each staining from the images in (**A**); scale bar represents 60 μm for all images; 40× objective.

Figure 3 shows a biopsy from mixed germ cell stromal tumour in a seven-year old male dog, where mixed tumour was found in testicle (based on morphological assessment), and also in the bladder. PAD2 and PAD4 were the most strongly detected PAD isozymes, CitH3 detection was high, while pan-deimination was detected at lower levels. PAD1 was detected in some nuclei, but also at some level in cytoplasm. PAD2 detection was strong in nucleus and cytoplasm. PAD3 showed nuclear localisation, also with some positive cytoplasmic staining, PAD4 staining was diffuse, PAD6 was positive in cytoplasm. CitH3 showed some diffuse staining but also nuclear staining. F95 staining was observed in cytoplasm.

**Figure 4:**
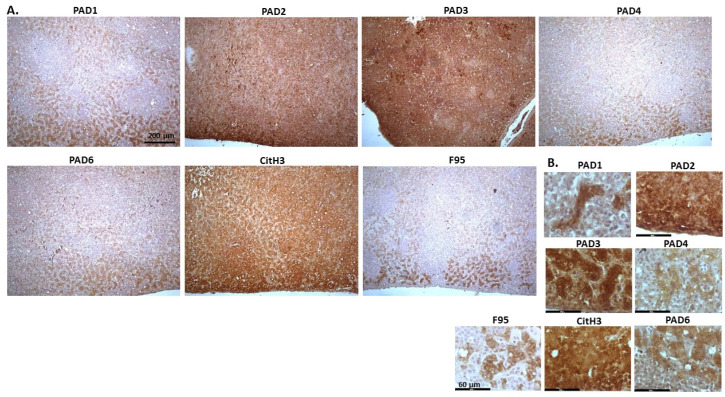
Regent parrot, lymphoma in liver and spleen. Cadaver, male bird. Tumour was observed in various organs, including liver, spleen, heart, kidney, adipose tissue and skin. Diagnosis: disseminated malignant round cell tumour, probable lymphoma. (**A**) Overview images for PAD1, 2, 3, 4, 6, CitH3 and F95 stainings of serial tissue sections; scale bar represents 200 μm for all images; 10× objective. (**B**) Higher magnification shown for each staining from the images in (**A**); scale bar represents 60 μm for all images; 40× objective.

Figure 4 shows lymphoma in Regent parrot, from a cadaver of a male bird. Tumour was found in various organs, including liver, spleen, heart, kidney, adipose tissue and skin and diagnosis was disseminated malignant round cell tumour, probable lymphoma. High levels of PAD2 and PAD3 were observed, as well as CitH3. Both PAD1 and PAD4 detection was low, some PAD6 levels were observed as well as pan-citrullination. PAD1 showed cytoplasmic stain, while PAD2 positive staining was observed in cytoplasm and nucleus; PAD3 positive staining was abundant in cytoplasm while nuclear staining was also observed. PAD4 staining was cytoplasmic; PAD6 staining was detected in cytoplasm and possibly some nuclear sites. CitH3 showed diffuse cytoplasmic, but also strong nuclear staining, while F95 positive staining was cytoplasmic.

**Figure 5:**
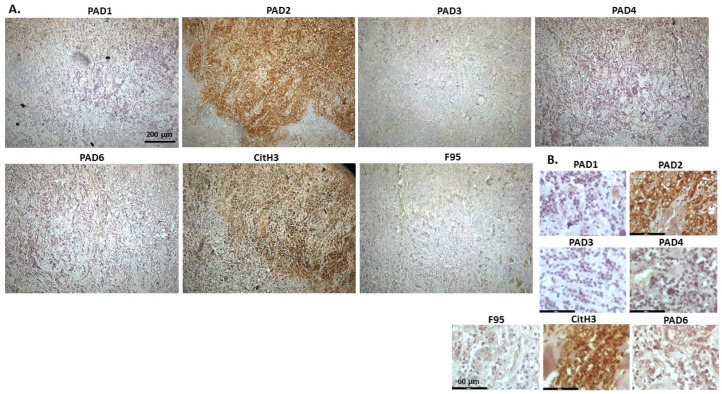
Sheep (two-year old ewe), malignant round cell tumour in skin. Origin of this highly malignant tumour is unknown. (**A**) Overview images for PAD1, 2, 3, 4, 6, CitH3, and F95 stainings of serial tissue sections; scale bar represents 200 μm for all images; 10× objective. (**B**) Higher magnification shown for each staining from the images in (**A**); scale bar represents 60 μm for all images; 40× objective.

Figure 5 shows malignant round cell tumour of sheep, from a two-year old ewe. Origin of the highly malignant tumour is unknown as only skin with tumour was received. PAD2 was observed to be the most strongly expressed PAD isozyme. CitH3 was detected at high levels, while total citrullination was low. The PAD2 staining observed was strongly detected in nucleus, while cytoplasmic staining was also clearly visible. PAD4 and PAD6 showed faint cytoplasmic stain. CitH3 showed nuclear localization, and some cytoplasmic stain, while diffuse positive cytoplasmic staining was observed for F95.

**Figure 6:**
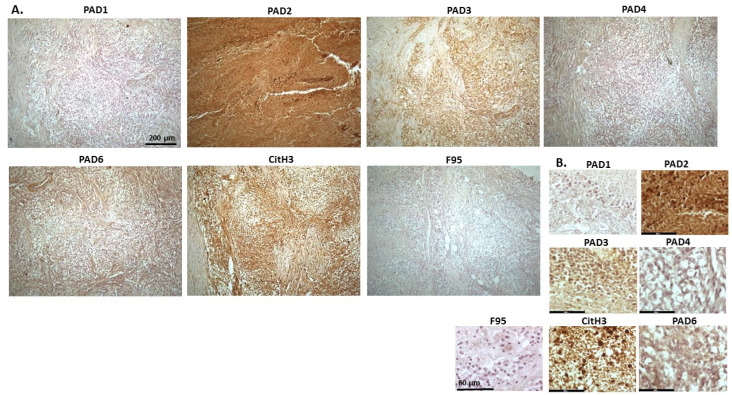
Mink, malignant renal tumour (possibly nephroblastoma). Cadaver, adult mink, male. Tumour in kidney with metastasis to lung and liver. (**A**) Overview images for PAD1, 2, 3, 4, 6, CitH3, and F95 stainings of serial tissue sections; scale bar represents 200 μm for all images; 10× objective. (**B**) Higher magnification shown for each staining from the images in (**A**); scale bar represents 60 μm for all images; 40× objective.

**Figure 7:**
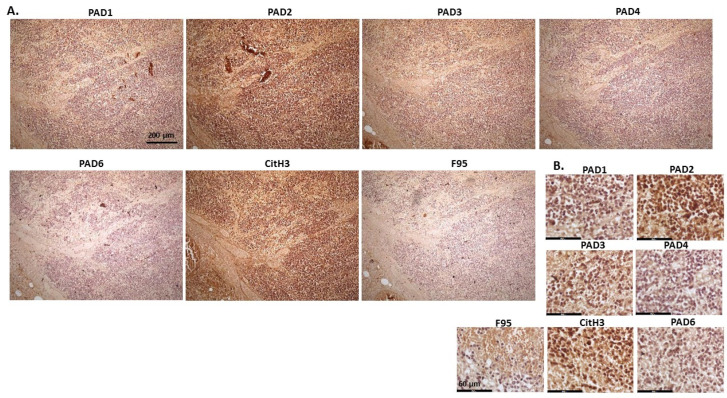
Mink, malignant renal tumour, probably nephroblastoma. Cadaver, mink, male. Extensive necrosis and haemorrhage in tumour mass. (**A**) Overview images for PAD1, 2, 3, 4, 6, CitH3, and F95 stainings of serial tissue sections; scale bar represents 200 μm for all images; 10× objective. (**B**) Higher magnification shown for each staining from the images in (**A**); scale bar represents 60 μm for all images; 40× objective.

Figure 6 and Figure 7 show representative images from malignant renal tumour of mink, possibly a nephroblastoma. The samples are from a cadaver of an adult male mink. Tumour was found in kidney with metastasis to lung and liver; the kidney is shown. The highest level of protein detection was seen for PAD2, with PAD3 and PAD6 also clearly detected, but PAD4 at lower levels. CitH3 detection was high, while total deimination levels were low. PAD1 staining was cytoplasmic, PAD2 showed strong positive, but diffuse staining, in cytoplasm, while some strong nuclear detection was also observed (Figure 6 and Figure 7). PAD3 showed some positive nuclear staining, and diffuse staining in cytoplasm. PAD4 stain was low and diffuse in cytoplasm (Figure 6) and also some nuclear localisation (Figure 7), while PAD6 showed mostly diffuse cytoplasmic, but also some evidence of nuclear staining (Figure 6 and Figure 7). CitH3 staining was nuclear (Figure 6 and Figure 7) and low level F95 staining was observed in cytoplasm (Figure 6), as well as some faint nuclear detection (Figure 7).

**Figure 8:**
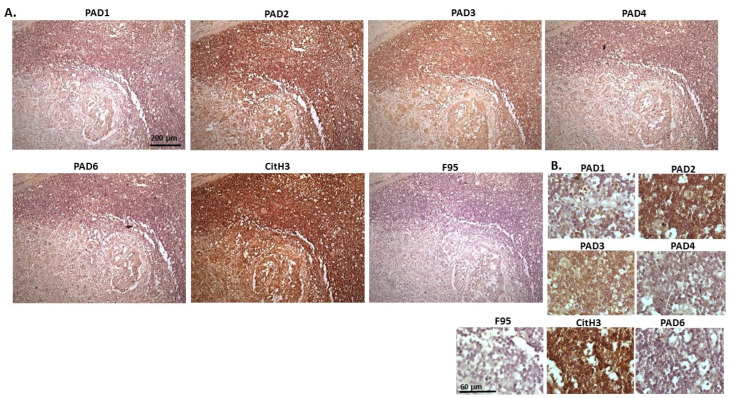
Cow, metastatic carcinoma, lungs and lymph nodes. Organ samples showed tumour in lungs and lymph nodes; origin of the primary tumour unknown. (**A**) Overview images for PAD1, 2, 3, 4, 6, CitH3 and F95 stainings of serial tissue sections; scale bar represents 200 μm for all images; 10× objective. (**B**) Higher magnification shown for each staining from the images in (**A**); scale bar represents 60 μm for all images; 40× objective.

Figure 8 shows metastatic carcinoma in lymph nodes of cow, with highest levels of PAD2, followed by PAD3 and high levels of CitH3. Pan-deimination was negligible. PAD1 positive stain was observed in some nuclear sites, and also some faint cytoplasmic stain. PAD2 positive staining was located to nucleus, as well as some cytoplasmic stain. PAD3 staining showed positive nuclear, as well as cytoplasmic. PAD4 positive staining was diffuse cytoplasmic, with nuclear detection observed in some sites. PAD6 positive staining was observed in occasional nuclear sites, but diffuse cytoplasmic staining was also detected. CitH3 staining was strongly nuclear, while low F95 staining was diffuse and cytoplasmic.

**Figure 9:**
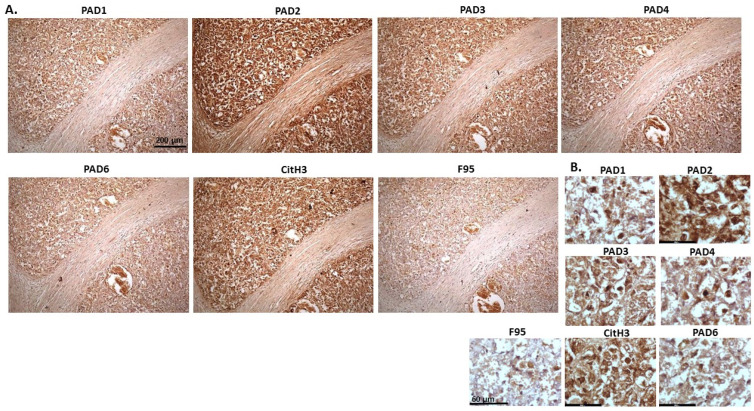
Deer, multicentric malignant tumour, probably mesothelioma. Organ samples from a female reindeer. Tumour masses in the abdominal cavity. Spleen and diaphragm were identified with tumour. (**A**) Overview images for PAD1, 2, 3, 4, 6, CitH3 and F95 stainings of serial tissue sections; scale bar represents 200 μm for all images; 10× objective. (**B**) Higher magnification shown for each staining from the images in (**A**); scale bar represents 60 μm for all images; 40× objective.

Figure 9 shows representative images from a multicentric malignant tumour, likely mesothelioma, of a female reindeer. Organ samples were taken and tumour masses found in the abdominal cavity. Spleen and diaphragm were identified with the tumour; representative images show strongest detection of PAD2, followed by PAD3 and PAD4, as well as some positive staining for PAD1 and PAD6. CitH3 detection was high and pan-deimination was also detected. PAD1 staining was localised to nucleus, as well as cytoplasm, PAD2 was strongly nuclear and cytoplasmic, PAD3 was strong in nucleus and cytoplasm was also positive. PAD4 staining showed positive in some nuclear sites and fainter cytoplasmic stain was also detected. PAD6 was localised to nuclei in places and cytoplasmic staining was also observed. CitH3 positive stain localised strongly to nucleus and F95 positive staining was cytoplasmic with some fainter occasional positive nuclei.

**Figure 10:**
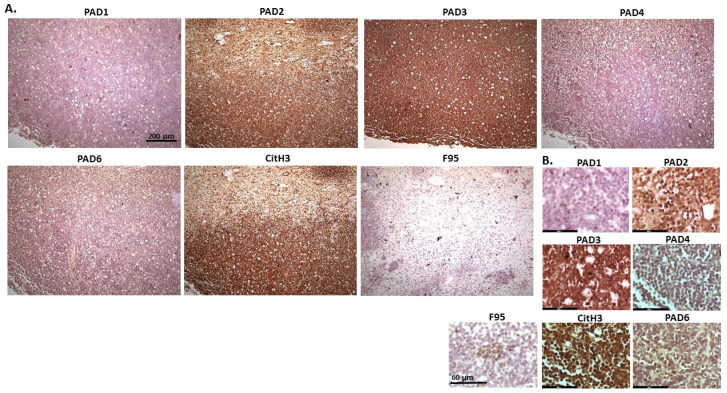
Pig, lymphoma (lymph node and kidney). (**A**) Overview images for PAD1, 2, 3, 4, 6, CitH3 and F95 stainings of serial tissue sections; scale bar represents 200 μm for all images; 10× objective. (**B**) Higher magnification shown for each staining from the images in (**A**); scale bar represents 60 μm for all images; 40× objective.

Figure 10 shows images of lymphoma in pig. Organ samples were taken from a slaughtered pig, with lymphoma observed in lymph node and kidney. Representative images show highest levels of PAD3, followed by PAD2, while detection of the other PAD isozymes was low. High detection was observed for CitH3 staining, while pan-deimination was negligible. PAD1 staining was overall negligible; PAD2 staining was strongly nuclear as well as cytoplasmic. PAD3 detection was strongly cytoplasmic, with also some positive nuclear detection, while PAD4 showed only occasional positive nuclear staining and some faint cytoplasmic staining. PAD6 positive staining was observed in occasional nuclei, and also some diffuse cytoplasmic stain. CitH3 was very strong in nuclei, while F95 positive staining was faint, with some positive nuclei (as well as some faint cytoplasmic stain).

**Figure 11:**
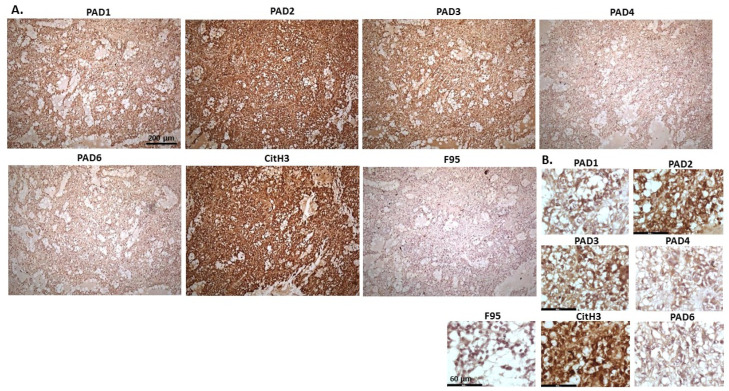
Dog Granulosa cell tumour, ovary; Samples from ovary of a five-year-old bitch. (**A**) Overview images for PAD1, 2, 3, 4, 6, CitH3, and F95 stainings of serial tissue sections; scale bar represents 200 μm for all images; 10× objective. (**B**) Higher magnification shown for each staining from the images in (**A**); scale bar represents 60 μm for all images; 40× objective.

Figure 11 represents images from canine granulosa cell tumour in ovary, with samples taken from the ovary of a five-year-old bitch. Highest detection of staining was seen for PAD2, followed by PAD3, with some positive staining for PAD1, while PAD4 and PAD6 were detected at low levels. CitH3 showed strong positive detection, while pan-deimination was low. PAD1 staining was clearly detected in nuclei, as well as positive in cytoplasm; PAD2 staining showed strong cytoplasmic detection and also nuclear detection; PAD3 staining showed positive in nuclei, also with positive detection in cytoplasm; PAD4 detection was overall lower with some positive staining seen in occasional nuclei and a diffuse positive cytoplasmic stain observed; a similar pattern was seen for PAD6. CitH3 positive staining was strongly nuclear, and also cytoplasmic, while diffuse F95 positive staining was observed mainly in cytoplasm.

**Figure 12:**
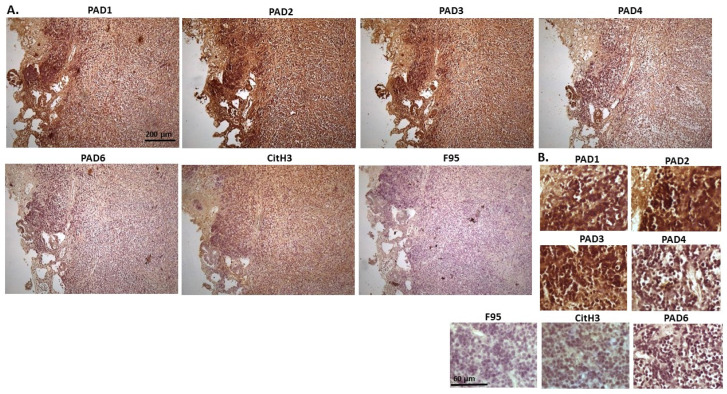
Hamster lymphoma, mesentery/small intestine. Cadaver of a hamster with tumour masses in mesentery. Suspected multicentric lymphoma cause by Hamster Polyomavirus (HaPV). (**A**) Overview images for PAD1, 2, 3, 4, 6, CitH3 and F95 stainings of serial tissue sections; scale bar represents 200 μm for all images; 10× objective. (**B**) Higher magnification shown for each staining from the images in (**A**); scale bar represents 60 μm for all images; 40× objective.

Figure 12 shows samples taken from a hamster cadaver with tumour masses detected in the mesentery/small intestine. This is a suspected multicentric lymphoma caused by Hamster Polyomavirus (HaPV). The highest levels were seen for PAD2 and PAD3 staining, as well as PAD1, while PAD4 and PAD6 showed lower positive. CitH3 detection was moderate while low levels for pan-deimination were observed. PAD1 staining was observed in nuclei as well as cytoplasm; PAD2 positive staining was strongly nuclear and also strongly detected in cytoplasm. PAD3 detection in the nucleus was strong, and positive staining was furthermore detected in the cytoplasm. PAD4 staining showed an overall diffuse cytoplasmic stain, with some positive nuclear staining, while PAD6 detection was low and diffuse in cytoplasm. CitH3 showed occasional positive in nuclei, but staining was lower than observed for many of the other tumours, while F95 detection could be seen at very low levels in cytoplasm, with occasional faint positive nuclei.

**Figure 13:**
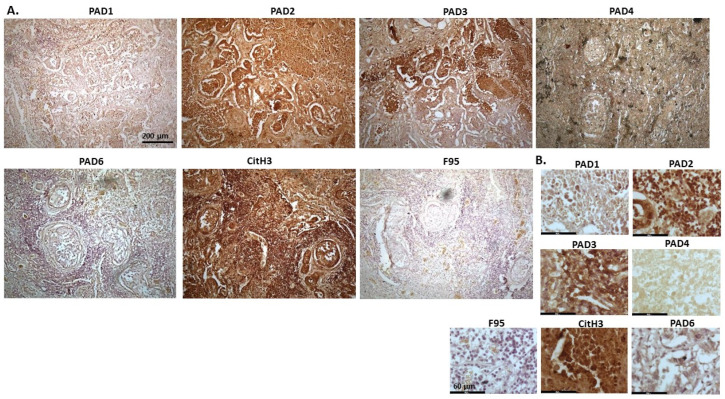
Cat, Pulmonary carcinoma (primary lung tumour suspected, with metastasis to heart); the sample is from a cadaver of 13-year old male cat. (**A**) Overview images for PAD1, 2, 3, 4, 6, CitH3 and F95 stainings of serial tissue sections; scale bar represents 200 μm for all images; 10× objective. (**B**) Higher magnification shown for each staining from the images in (**A**); scale bar represents 60 μm for all images; 40× objective.

Figure 13 shows pulmonary carcinoma from the cadaver of 13-year old male cat, with metastasis to the heart. This is a suspected primary lung tumour, as no other primary tumour was found (although this is rare in animals). Representative images show a strong detection for PAD2, followed by PAD3, while PAD4 also showed some lower positive, and PAD1 and PAD6 both showed low positive. A very strong positive staining was seen for CitH3, while pan-deimination (F95) detection was low. PAD1 staining was observed in some nuclei, with some cytoplasmic detection observed as well. PAD2 staining was strongly cytoplasmic with positive detection in nuclei. PAD3 showed some strong nuclear detection and diffuse cytoplasmic staining, while PAD4 staining was diffuse and cytoplasmic. PAD6 was cytoplasmic, with some faint positive nuclei. CitH3 staining was strongly nuclear, with positive cytoplasmic staining as well, while some diffuse F95 positive staining could be observed.

**Figure 14:**
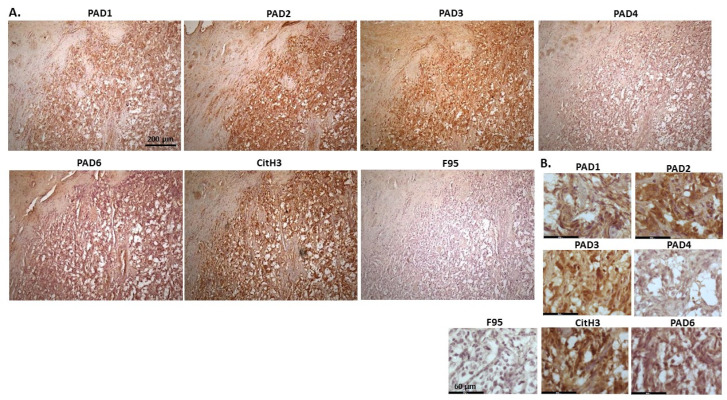
Cow, tumour metastasis to the heart. Origin of the tumour is uncertain but can be from chemoreceptors at the carotid or aortic body, from C-cells in the thyroid gland (ultimobranchial tumour or adrenal gland medulla), possible pheochromocytoma—neuroendocrine tumour. (**A**) Overview images for PAD1, 2, 3, 4, 6, CitH3 and F95 stainings of serial tissue sections; scale bar represents 200 μm for all images; 10× objective. (**B**) Higher magnification shown for each staining from the images in (**A**); scale bar represents 60 μm for all images; 40× objective.

Figure 14 shows representative images of a tumour observed on the right heart ventricle of a cow. The origin of the tumour is uncertain and could be from chemoreceptors at the carotid or aortic body, from C-cells in the thyroid gland (ultimobranchial tumour) or adrenal gland medulla (pheochromocytoma—neuroendocrine tumour). PAD2 and PAD3 staining showed the highest detection of the PAD isozymes, followed by PAD1. PAD4 positive staining was low, while PAD6 staining was clearly detected. Furthermore, CitH3 detection was high but pan-deimination was low. PAD1 positive detection was observed mainly in cytoplasm, with occasional nuclear staining. PAD2 was strongly cytoplasmic but also localised to some nuclei. PAD3 staining was strong in nuclei, with some positive detection in cytoplasm, while PAD4 positive detection was diffuse and cytoplasmic. PAD6 was detected in some nuclei alongside diffuse cytoplasmic staining. CitH3 detection was high in nuclei while F95 staining was low and diffuse in the cytoplasm.

**Figure 15:**
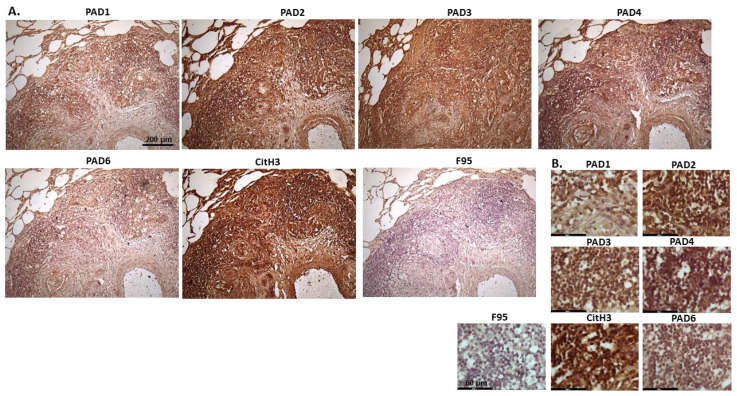
Cow, metastatic carcinoma, lungs and lymph nodes. Tumour in lungs and lymph nodes; origin of the primary tumour unknown. (**A**) Overview images for PAD1, 2, 3, 4, 6, CitH3 and F95 stainings of serial tissue sections; scale bar represents 200 μm for all images; 10× objective. (**B**) Higher magnification shown for each staining from the images in (**A**); scale bar represents 60 μm for all images; 40× objective.

Figure 15 shows metastatic carcinoma of unknown origin detected in lungs and lymph nodes of cow. The strongest positive staining was observed for PAD2 and PAD3, while PAD4 was also detected at high levels, but PAD1 and PAD6 at lower levels. CitH3 staining was very high, but pan-deimination low. PAD1 showed diffuse cytoplasmic staining, with some evidence of nuclear localisation. PAD2 showed strong cytoplasmic staining and some strong nuclear staining was also observed. PAD3 showed strong cytoplasmic staining, with some nuclear detection, while PAD4 staining was both cytoplasmic, as well as with occasional positive nuclei. PAD6 showed mainly diffuse cytoplasmic staining. CitH3 staining was strongly nuclear, while faint and diffuse F95 positive cytoplasmic staining was observed.

**Figure 16:**
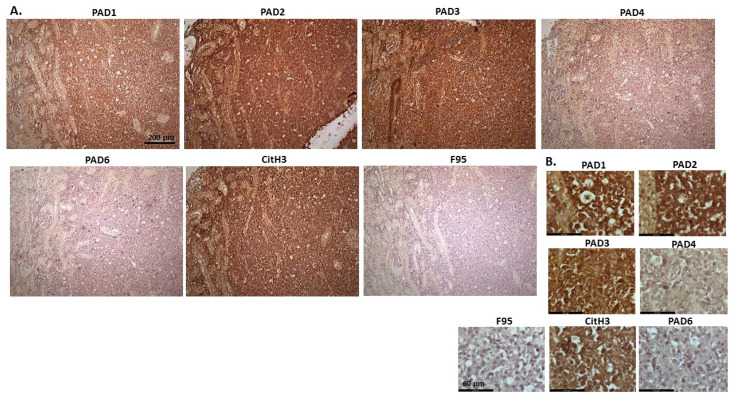
Cat, malignant tumour in kidneys; probably lymphoma. (**A**) Overview images for PAD1, 2, 3, 4, 6, CitH3, and F95 stainings of serial tissue sections; scale bar represents 200 μm for all images; 10× objective. (B) Higher magnification shown for each staining from the images in (**A**); scale bar represents 60 μm for all images; 40× objective.

Figure 16 shows malignant tumour, probably lymphoma, in the kidney of an eight-month old male cat. A tumour was observed in both kidneys. PAD2 and PAD3 positive staining was detected at very high levels, followed by PAD1, but PAD4 staining was detected at low levels and PAD6 staining was also low. CitH3 detection was very high, while pan-deimination detection was negligible. PAD1 positive staining showed both some nuclear localisation as well as cytoplasmic detection, while PAD2 was very strongly cytoplasmic, but also with positive nuclear detection. PAD3 showed strong detection in nucleus and was also clearly observed in cytoplasm. PAD4 positive staining was detected in cytoplasm and low staining in occasional nuclei. CitH3 staining was strongly nuclear but F95 staining very faint cytoplasmic.

**Figure 17:**
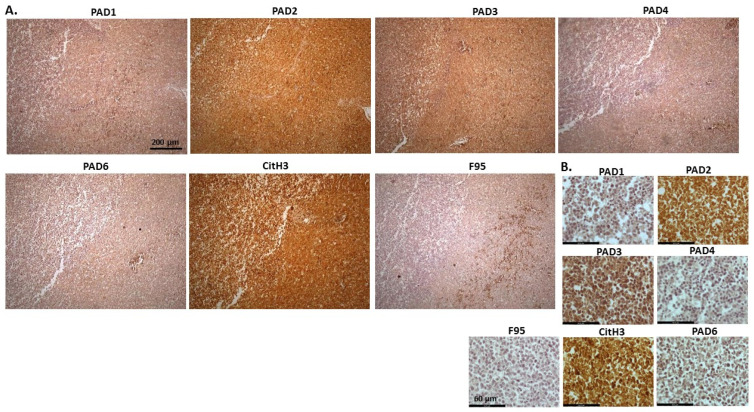
Domestic duck, malignant round cell tumour, liver; probably lymphoma. (**A**) Overview images for PAD1, 2, 3, 4, 6, CitH3, and F95 stainings of serial tissue sections; scale bar represents 200 μm for all images; 10× objective. (**B**) Higher magnification shown for each staining from the images in (**A**); scale bar represents 60 μm for all images; 40× objective.

Figure 17 shows malignant round cell tumour (probably lymphoma) in the liver of domestic duck. The highest level of protein detection was observed for PAD2, followed by PAD3. PAD1 and PAD4 were also detectable at lower levels. CitH3 detection was high. Total deimination was also detected, albeit low. PAD1 staining was diffuse and mainly cytoplasmic, with some occasional nuclear detection. PAD2 positive staining was strongly cytoplasmic, also with nuclear detection, and a similar pattern was observed for PAD3, albeit at lower levels than for PAD2. PAD4 showed diffuse cytoplasmic staining; PAD6 showed some positive detection in nuclei, as well as diffuse cytoplasmic staining. CitH3 was strongly positive in nuclei and some diffuse cytoplasmic staining was observed for F95.

**Figure 18:**
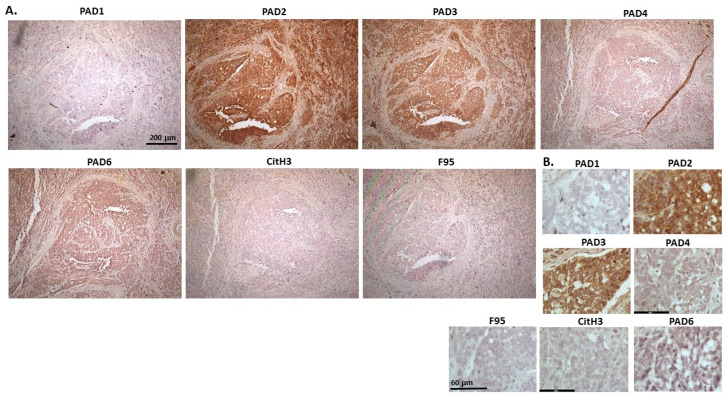
Horse (18-year-old mare), with carcinoma in liver, ovary and mesenteric lymph node. Carcinoma in liver is probable primary bile duct carcinoma but tumour metastasis to the liver cannot be ruled out. (**A**) Overview images for PAD1, 2, 3, 4, 6, CitH3, and F95 stainings of serial tissue sections; scale bar represents 200 μm for all images; 10× objective. (**B**) Higher magnification shown for each staining from the images in (**A**); scale bar represents 60 μm for all images; 40× objective.

Figure 18 shows carcinoma in horse, which was detected in the liver, ovary, and mesenteric lymph nodes. Organs were taken from an 18-year-old mare and carcinoma found in liver; probable primary bile duct carcinoma but tumour metastasis to the liver cannot be ruled out. There was high detection of PAD2 positive staining, followed by PAD3 and PAD6, while PAD4 staining was very low and PAD1 staining negligible. CitH3 and F95 staining was also negligible. PAD2 positive staining was strongly cytoplasmic, with some nuclear staining also observed. PAD3 staining was cytoplasmic, with some occasional nuclear detection, while both PAD4 and PAD6 showed some diffuse positive cytoplasmic staining.

**Figure 19:**
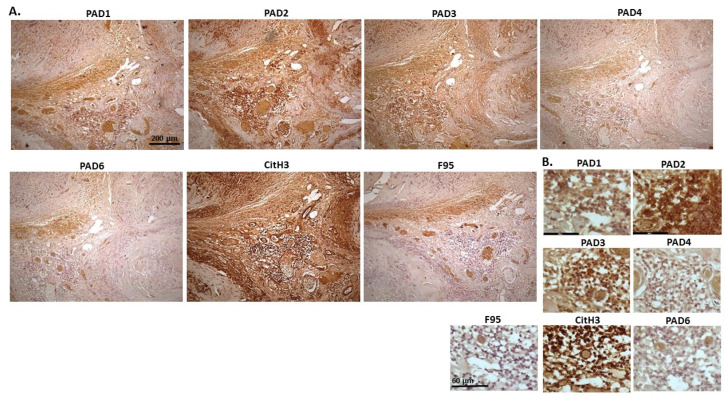
Dog; biopsy from a tumour in mammary gland of an eight-year-old bitch. Scirrhous adenocarcinoma in mammary gland. (**A**) Overview images for PAD1, 2, 3, 4, 6, CitH3 and F95 stainings of serial tissue sections; scale bar represents 200 μm for all images; 10× objective. (**B**) Higher magnification shown for each staining from the images in (**A**); scale bar represents 60 μm for all images; 40× objective.

Figure 19 shows a sample of a biopsy from a tumour in the mammary gland of an eight-year-old bitch. This was identified as scirrhous adenocarcinoma. PAD2 showed the highest positive staining levels of the PAD isozymes, followed by PAD3 and PAD1, while PAD4 and PAD6 staining was low. A very strong positive staining was seen for CitH3, and some positive staining was also observed for pan-deimination. PAD1 staining was cytoplasmic, with occasional positive nuclei, while PAD2 positive detection was observed clearly in nuclei, as well as in cytoplasm. PAD3 showed positive staining in cytoplasm and some nuclei, while PAD4 showed positive for occasional nuclei and diffuse cytoplasmic staining. PAD6 showed diffuse positive cytoplasmic staining. CitH3 was strongly detected in nuclei and also some cytoplasmic staining, while F95 positive staining was low and diffuse in cytoplasm.

**Figure 20:**
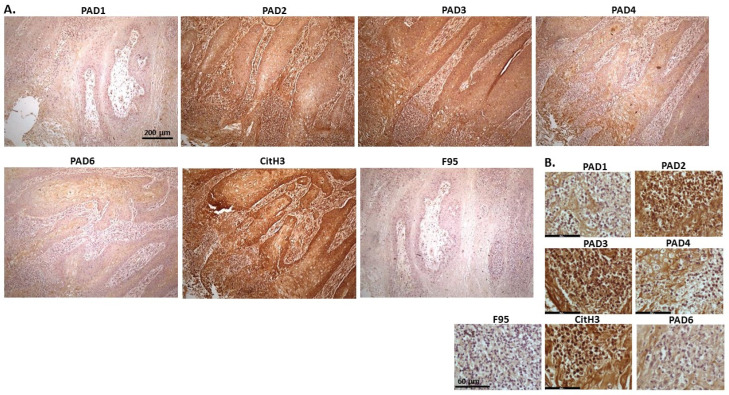
Horse, squamous penile cell carcinoma, papilloma. Scale bar represents 200 μm for all images. (**A**) Overview images for PAD1, 2, 3, 4, 6, CitH3, and F95 stainings of serial tissue sections; scale bar represents 200 μm for all images; 10× objective. (**B**) Higher magnification shown for each staining from the images in (**A**); scale bar represents 60 μm for all images; 40× objective.

Figure 20 shows penile squamous cell carcinoma of a horse. Both PAD2 and PAD3 were the most strongly detected isozymes, followed by PAD4. Some positive staining was observed also for PAD1 and PAD6. CitH3 staining was very strong, while pan-deimination detection was low. PAD1 positive staining was cytoplasmic, while PAD2 and PAD3 staining was strongly detected in cytoplasm as well as in nuclei. PAD4 staining was mainly cytoplasmic and PAD6 showed also positive cytoplasmic staining. CitH3 staining showed strongly positive in nuclei, and also in cytoplasm, while diffuse faint cytoplasmic F95 staining was observed.

**Figure 21:**
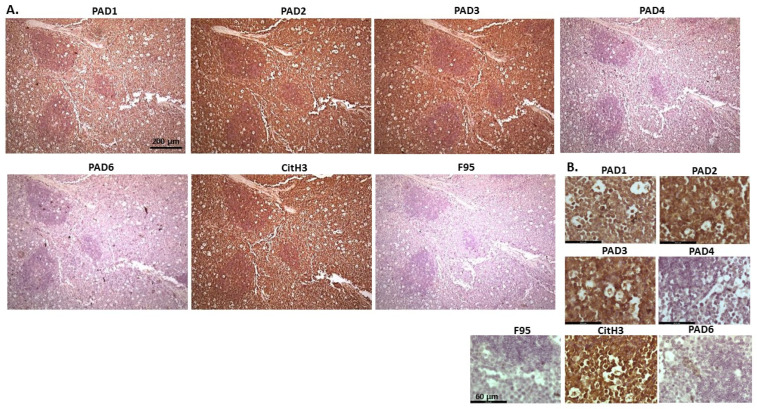
Cow—lymphoma. Organ samples from a cow kidney and lymph nodes were analysed. (**A**) Overview images for PAD1, 2, 3, 4, 6, CitH3, and F95 stainings of serial tissue sections; scale bar represents 200 μm for all images; 10× objective. (**B**) Higher magnification shown for each staining from the images in (**A**); scale bar represents 60 μm for all images; 40× objective.

Figure 21 shows representative images of cow lymphoma. Organ samples taken included kidney and lymph nodes. PAD2 and PAD3 positive staining was found to be the highest out of the PAD isozymes, PAD1 staining was also positive, but PAD4 and PAD6 staining was negligible. CitH3 staining was detected at very high levels, while pan-deimination was very low. PAD1 staining showed positive in cytoplasm, but also some occasional nuclear staining, while PAD2 and PAD3 staining was strongly cytoplasmic, also with some nuclear detection. CitH3 showed strong positive nuclear staining, and cytoplasmic staining was also observed, while F95 staining was low and diffuse in cytoplasm.

### 2.2. Phylogeny Tree Construction for PAD Isozymes in the Species under Study

**Figure 22:**
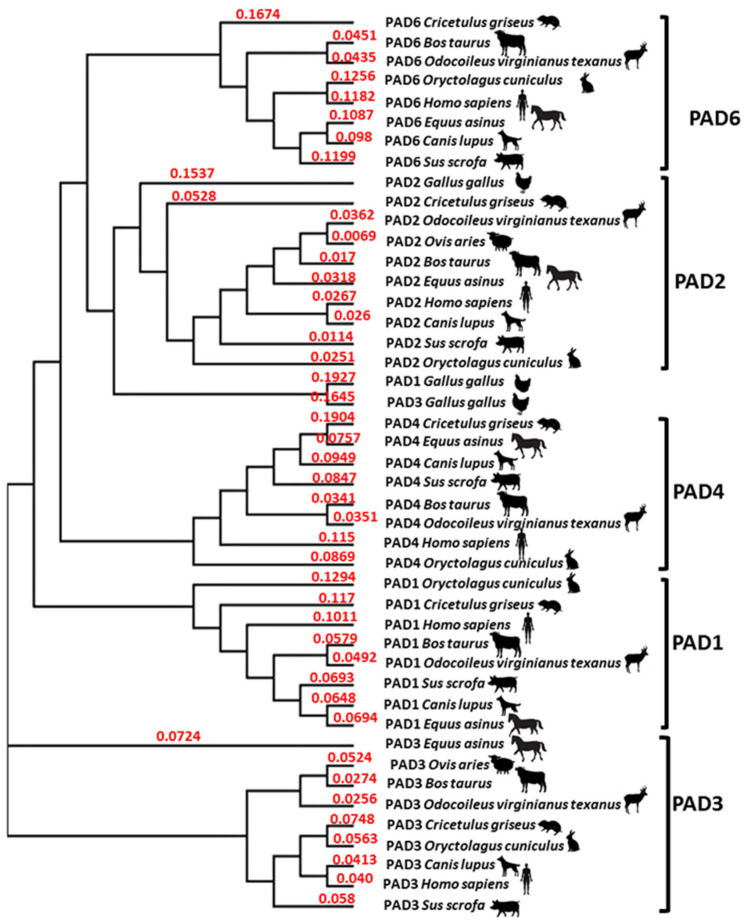
Neighbour joining tree for PAD isoforms. PAD isozymes from the species under study, or representative species if species-specific sequences were not available in NCBI, are included in the phylogeny tree in comparison with human PADs. The different PAD isoforms do group together in clades, except for the PADs from Aves, with all three known PAD isozymes (PAD1, 2, 3; using chicken, *Gallus gallus*, as a representative species), grouping closest with the PAD2 clade. The following protein sequences from NCBI were used: Human (*Homo sapiens* NP_037490.2_PAD1, NP_031391.2_PAD2, NP_057317.2_PAD3, NP_036519.2_PAD4, AAR38850.1_PAD6); Chicken (as a representative for birds; *Gallus gallus* NP_001305368.1_PAD1, NP_001305948.1_PAD2, NP_990374.2_PAD3); Rabbit (*Oryctolagus cuniculus* XP_017194808.1_PAD1, XP_002723905.1_PAD2, XP_017194809.1_PAD3, XP_008249780.1_PAD4, XP_008249781.1_PAD6); Hamster, using *Cricetulus griseus* as a representative species (EGW12396.1_PAD1, ERE81130.1_PAD2, XP_003514861.1_PAD3, EGW12398.1_PAD4, EGV98983.1_PAD6); Dog (*Canis lupus* XP_851932.1_PAD1, XP_038516105.1_PAD2, XP_038516102.1_PAD3, XP_848494.1_PAD4, NP_001091016.3_PAD6); Horse, using *Equus asinus* as a representative species (XP_014691666.1_PAD1, XP_014691667.1_PAD2, XP_014691664.1_PAD3, XP_014691660.1_PAD4, XP_014691633.1_PAD6); Cow (*Bos taurus* NP_001094742.1_PAD1, XP_010800917.1_PAD2, XP_010800991.1_PAD3, NP_001179102.1_PAD4, XP_002685843.1_PAD6); Sheep (*Ovis aries*—only PAD2 and PAD3 were available in NCBI: XP_027821466.1_PAD2, NP_001009791.1_PAD3); Deer, using white tailed deer as a representative species (*Odocoileus virginianus texanus* XP_020733655.1_PAD1, XP_020733656.1_PAD2, XP_020733658.1_PAD3, XP_020754850.1_PAD4, XP_020754849.1_PAD6); Pig (*Sus scrofa* XP_003127695.3_PAD1, JAA53543.1_PAD2, XP_003127694.1_PAD3, XP_003127696.1_PAD4, XP_013854479.1_PAD6). No published PAD sequences for mink were found and it is therefore not included in the tree. The red numbers represent a measure of support for the node.

A phylogeny tree was created for the PAD isozymes present in the representative species under study here. This confirmed that vertebrata PADs group with human PAD isozymes. The three PAD isozymes present in Aves, which here are represented by *Gallus gallus* PAD1, 2 and 3, all grouped closely together with the vertebrate PAD2 clade (Figure 22).

## 3. Discussion

The current study explored peptidylarginine deiminase (PAD) isozyme protein detection and the presence of deiminated/citrullinated protein products in a range of cancers across the animal kingdom. The cancers chosen for this pilot analysis included mammary, kidney, lung, testicular, neuroendocrine, and anaplastic cancers; papilloma, lymphoma and granulosa cell tumour; from a range of vertebrate species. The findings of this pilot study indicate that histone H3 deimination (CitH3) was strongly detected in almost all of the animal cancers assessed, pan-deimination detection as assessed by F95 reactivity was overall lower, and staining intensity of the five PAD isozymes varied between cancer types, with PAD2 and PAD3 being the most prominent isozymes across the specimens assessed.

Immunohistochemical analysis of archived histological tissue sections was performed for comparative assessment of PAD1, PAD2, PAD3, PAD4, and PAD6 isozyme detection, via cross-reaction using commercially available anti-human antibodies. This was carried out due to the lack of commercial availability of species-specific antibodies and these antibodies have furthermore previously been verified for cross-reaction across a range of animal species. In addition, total protein deimination was assessed, using the commercially available pan-citrulline/deimination F95 antibody (initially published in [35]) and histone H3 deimination was specifically assessed, using the commercially available CitH3 (citrulline R2 + R8 + R17) antibody. It must be emphasized that the current study is limited to the sole use of immunohistochemistry and associated intensity scoring of staining (as shown in Figure 1), while further methods for quantification were not included here. Based on the findings presented here from the immunohistochemical staining, CitH3 was overall very strongly detected in all of the animal cancers assessed (with the exception of equine liver carcinoma). This aligns with previous literature reporting increase in histone citrullination associated with various cancers. Importantly, histone H3 deimination is indicative of PAD-mediated gene regulatory changes and is furthermore a marker of NETosis; the formation of neutrophil extracellular traps in response to pathogens and inflammatory conditions, including with strong association to cancer-related processes [36,37]. NET formation as identified by immunohistochemistry against histone citrullination has for example been verified in Hodgkin’s lymphoma, indicative of contribution to the inflammatory tumour microenvironment [38]. Anti-cyclic citrullinated peptide antibodies have furthermore been assessed in relation to lymphoma risk in RA patients, but no significant correlation was found in one study [39], while in diffuse large B-cell lymphoma, a higher prevalence of antibodies against citrullinated proteins was identified, although it was not a prognostic marker [40]. Importantly, CitH3 has been identified as a strong pro-oncogenic marker in a range of human cancers, including as a circulatory biomarker [1].

For detection of pan-citrullination, F95 positive staining was overall faint in all of the samples assessed, but showed some significance in malignant round cell tumour of duck, lymphoma of parrot, scirrhous adenocarcinoma and mixed germ cell-sex cord stromal tumour of dog, multicentric malignant tumour of deer, and mink nephroblastoma and mammary adenocarcinoma in rabbit. Therefore, while F95 detection would potentially identify all deiminated proteins [35], this may be at lower levels than the CitH3 antibody, which specifically identifies histone H3 deimination.

While PAD isozyme detection levels varied somewhat between the different cancer types in the different species, PAD2 and PAD3 were the most predominant isozymes detected at high levels. While such variation may in part be attributed to, and representative of, different isozyme presence/dominance between species, as well as reflect tissue specific expression, these findings indicate a significant role for these two PAD isozymes in cancer. PAD2, which is the PAD considered ancestral and most ubiquitously expressed PAD in mammals, was found at highest levels overall across the cancers, and interestingly, PAD3 was also found at high levels in many of the cancers assessed in the different animal species. This correlates with recent findings that PAD3 may be linked to more aggressive types of cancers, including glioblastoma and pancreatic cancer [19,20]. As PAD3 is also a stem-cell related PAD [17] a role for PAD3 in aggressive cancers is of considerable interest and warrants further investigation. Interestingly, PAD4, which has been much researched in cancers, did not show consistently high expression in the cancers assessed in this current pilot study. It may though also need to be considered that PAD4 down-regulation can be associated with some cancers, rather than expecting PAD4 increase, as indeed in human cancers mutations and deletions in the PAD gene locus region are reported [41,42]. PAD expression is though generally reported as increased in tumours, with PAD2 and PAD4 reported to be the most commonly expressed PAD isozymes and PAD4 overexpression was reported in carcinomas of kidney, soft tissue tumours, oesophagus, liver, lung, colon, gastric, bladder, breast and ovary, while PAD2 overexpression was reported in small lung cell and prostate cancers, and also in breast cancer [29,43,44,45,46,47,48]. Nevertheless, PAD2 downregulation has though also been reported in some cancers, for example downregulation of PAD2 has been found to be an early event in colorectal carcinogenesis [30].

Previous studies on PADs in animal cancers are scarce, and have mainly focused on mammary tumours, assessing PAD2 expression in canine and feline mammary tumours, also in comparison to human breast cancer [44]. Furthermore, pan-citrullination and histone H3 citrullination have been reported in canine mammary epithelial cells during diestrus [33] and the citrullinome of lactating mouse mammary gland has been reported, pointing to roles in gene expression and microtubule dynamics [49]. Interestingly, previous reports have shown that while normal human and canine mammary epithelium display high PAD2 expression, PAD2 is reduced in carcinomas in both canine and human, and in feline, a complete loss of nuclear PAD2 expression was observed. Therefore, it has been suggested that loss of nuclear PAD2 expression may be representative of more aggressive neoplasms [31]. The roles of PAD2-mediated function in various cancers are complex, including via regulation of transcription in cancer progression [50]. The use of PAD inhibitor BB-Cl-amidine has been assessed for PAD2 and PAD4 expression and shown to reduce viability and tumourigenicity of feline and canine mammary cancer lines by affecting ER stress, and furthermore tested in xenograft models [34].

An interesting observation in the current study is the detection of PAD6, often at considerably high levels, in some of the animal cancers assessed. While PAD6 is, according to published studies, mainly linked to developmental processes including embryonic development, oocyte formation and embryo implantation, its role in cancers has received little attention to date. No link to cancer has been published to our knowledge, besides a genome-based SNP (single nucleotide polymorphism) connection in Icelandic individuals demonstrating a considerable link of cutaneous-basal cell carcinoma possibilities and mutation amongst PAD6 and PAD4 locus within 1p36 [51]. In the current study, PAD6 positive staining was observed at varying levels in a number of the animal cancers, including in rabbit mammary adenocarcinoma, parrot lymphoma, mink nephroblastoma, feline pulmonary carcinoma, equine liver carcinoma, bovine neuroendocrine tumour, multicentric malignant tumour (mesothelioma) of deer, pig lymphoma, canine anaplastic carcinoma and mixed germ cell-sex cord stromal tumour. It may be postulated that due to the developmental roles of PAD6 it could indeed be an important player in cancer progression and therefore have hitherto unrecognised roles in neoplasms, cancer aggressiveness and cancer evolution. Interestingly, in mouse knockout-models for PAD2 and PAD4 expression, upregulation of PAD6 was observed [52], which could possibly indicate that other PAD isozymes may take over function of redundant PAD isozymes in physiological and pathological processes.

It must furthermore be considered that the different PAD isozymes have preferences for different target proteins [53], and therefore the difference detected in PAD isozyme protein levels in the different cancers here, may possibly result in deimination of different pro-cancerous proteins. This will need further evaluation, including via larger scale proteomic analysis, and remains subject to further studies. With respect to histone deimination, while only PAD4 has a classical nuclear translocation signal, both PAD2 and PAD3 are also reported with nuclear location and found to deiminate histones [11,16,17,31,33]. Histone citrullination is strongly associated to tumours [54] and CitH3 is a common marker for NETosis, which has also been used as an indicative marker for cancer-associated inflammatory processes. For example, such processes have been associated with awakening dormant cancer cells [55]. Furthermore, NETs are linked to cancer development and progression, as well as metastasis [56,57,58]. Overall, the high level of CitH3 positive detection in the animal cancers examined in the current study indicates an important role for histone H3 deimination. While all five PADs are expressed in mammals, birds only have three PAD isozymes, all of which align the closest with the Vertebrata PAD2 clade. This correlates with the current finding that PAD2 and PAD3 showed strongest detection for the PADs in the bird cancers assessed. Furthermore, the strong detection of CitH3 in both bird cancers assessed supports that PAD2 and PAD3 may be responsible for histone H3 deimination in Aves.

In summary, the findings of this pilot study may provide novel insights into PAD-mediated roles in different cancers across a range of mammalian and avian species and may aid furthering understanding of PADs in cancer evolution.

## 4. Materials and Methods

### 4.1. Tissue Sections from Animal Cancers

Paraffin embedded archived tissues from a range of animal cancers were used for the study, collected during routine appointments at the Institute for Experimental Pathology, University of Iceland (under licence number #0002 kt-650269-4549), approved by the central animal ethics committee in Iceland (Icelandic Food Regulation Authority, MAST Matvælastofnun). The samples were confirmed by a veterinary pathologist (Institute for Experimental Pathology, University of Iceland). Samples were fixed according to standard procedures in 4% PFA, followed by embedding in paraffin and the tissue blocks stored at room temperature until sectioned. Cancers selected for assessment in the current study were from a range of vertebrate species as follows: horse (*Equus caballus*), cow (*Bos taurus*), reindeer (*Rangifer tarandus*), pig (*Sus scrofa*), sheep (*Ovis aries*), dog (*Canis lupus familiaris*), cat (*Felis catus*), mink (*Neovision vision*), rabbit (*Oryctolagus cuniculus*), hamster (*Mesocricetus auratus)*, Regent parrot (*Polytelis anthopeplus*) and domestic duck (*Anas platyrhynchos*). Table 2 provides an overview of the histological samples used in the current pilot study, including information on species, cancer type, and histology sample type.

### 4.2. Immunohistochemical Analysis

Tissue sections (5 μm serial sections) were deparaffinised and stained according to previously established protocols for the detection of pan-citrullination (F95 pan-citrulline/deimination antibody, MABN328, Merck, Feltham, UK), for citrullinated histone H3 (CitH3; ab5103, Abcam, Cambridge, UK) and for the five PAD isozymes PAD1, 2, 3, 4, and 6, according to methods described in: [5,16,59]. In summary, the tissue sections were deparaffinised in xylene (3 × 10 min), followed by immersion in 100% isopropanol for 5 min and sequential incubation in ethanol (100, 90% and 70%), 5 min each, and thereafter the sections were taken to water (dH_2_O). Antigen retrieval was performed by microwaving the sections in citric acid buffer (pH 6.0) for 12.5 min, at power 8. The sections were then allowed to cool at room temperature (RT) and taken to distilled water. Blocking was carried out using 5% goat serum (Sigma, St. Louis, MO, USA) in phosphate buffer (PB) for 1 h, followed by overnight incubation with the primary antibodies (all diluted 1/100 in 0.1% BSA) at 4 °C using the following primary antibodies: pan-citrulline/deimination F95 antibody (MABN328, Merck, Feltham, UK; original concentration 1 μg/μL), anti-human PAD1 (ab181762, Abcam; original concentration 1 μg/μL), anti-human PAD2 (ab50257), anti-human PAD3 (ab50246; original concentration 1 μg/μL), anti-human PAD4 (ab50247; original concentration 1 μg/μL) and anti-human PAD6 (PA5-72059, Thermo Fisher Scientific, Dartford, UK; original concentration 0.5 μg/μL); as well as the anti-histone H3 citrullination antibody (anti-CitH3, ab5103, Abcam, original concentration 1 μg/μL). Washing was performed in 100 mM PB, followed by secondary antibody incubation using anti-rabbit IgG or anti-mouse IgM biotinylated antibodies (Vector laboratories, Peterborough, UK; diluted 1/200) for 1 h, at RT. This was followed by amplification with Avidin-Biotinylated peroxidase Complex (ABC, Vector Laboratories) incubation for 1 h at RT; colour development was carried out using diaminobenzidine/hydrogen peroxide (DAB) stain for 5 min at RT. Background staining was performed using Mayer’s haematoxylin (Sigma, Gillingham, UK). Sections were dehydrated in alcohol, immersed in xylene, mounted with DEPEX (Sigma) and cover slipped. An overall labelling intensity score was determined, ranging from 0–3, with 0 as no labelling, 1 (+) as weak labelling (indicated in brackets if very weak), 2 (++) moderate labelling and 3 (+++) as strong labelling (see scoring index key in Figure 1). Digital images were captured on a Leica microscope, using the 10× or 40× objective, respectively, and a Sony AVT-Horn 3CCD colour video camera (24 bit RGB, 760 × 570 pixel resolution); representative scale bars were applied to the images.

### 4.3. Phylogenetic Tree Construction of PAD Isozymes in the Animal Species under Study

PAD isoforms from the animal species under study were used based on available protein sequences in NCBI, in some cases using a representative animal species if the species-specific PAD sequences were not available in NCBI. Sequence alignment for the PAD isoforms, also compared with human PAD isozymes, was carried out using Clustal Omega (https://www.ebi.ac.uk/Tools/msa/clustalo/ (accessed on 2 April 2022)) and thereafter a phylogeny tree was constructed in Clustal Omega. Sequences used for the analysis were the following: Human (*Homo sapiens*: NP_037490.2_PAD1; NP_031391.2_PAD2; NP_057317.2_PAD3; NP_036519.2_PAD4; AAR38850.1_PAD6); as a representative species for birds chicken (*Gallus gallus*) was used (NP_001305368.1_PAD1; NP_001305948.1_PAD2; NP_990374.2_PAD3); Rabbit (*Oryctolagus cuniculus*: XP_017194808.1_PAD1; XP_002723905.1_PAD2; XP_017194809.1_PAD3; XP_008249780.1_PAD4; XP_008249781.1_PAD6); Hamster, using *Cricetulus griseus* as a representative species (EGW12396.1_PAD1; ERE81130.1_PAD2; XP_003514861.1_PAD3; EGW12398.1_PAD4; EGV98983.1_PAD6); Dog (*Canis lupus*: XP_851932.1_PAD1; XP_038516105.1_PAD2; XP_038516102.1_PAD3; XP_848494.1_PAD4; NP_001091016.3_PAD6); Horse, using *Equus asinus* as a representative species (XP_014691666.1_PAD1; XP_014691667.1_PAD2; XP_014691664.1_PAD3; XP_014691660.1_PAD4; XP_014691633.1_PAD6); Cow (*Bos taurus*: NP_001094742.1_PAD1; XP_010800917.1_PAD2; XP_010800991.1_PAD3; NP_001179102.1_PAD4; XP_002685843.1_PAD6); Sheep (*Ovis aries*: only PAD2 and PAD3 were available in NCBI: XP_027821466.1_PAD2; NP_001009791.1_PAD3); Deer, using white tailed deer (*Odocoileus virginianus texanus*) as a representative species (XP_020733655.1_PAD1; XP_020733656.1_PAD2; XP_020733658.1_PAD3; XP_020754850.1_PAD4; XP_020754849.1_PAD6); Pig (*Sus scrofa*: XP_003127695.3_PAD1; JAA53543.1_PAD2; XP_003127694.1_PAD3; XP_003127696.1_PAD4; XP_013854479.1_PAD6). No published PAD sequences for mink were found in NCBI and mink is therefore not included in the phylogeny tree.

## 5. Conclusions

This is the first study to assess all five PAD isozymes in a selection of cancers across a wide range of vertebrate animal species, including mammals and birds. Although due to lack of species-specific antibodies this study relied on cross-reaction with anti-human PAD isozyme specific antibodies, these have previously been validated to cross-react with a number of other species. The findings of this pilot study indicate that histone H3 deimination (CitH3) was strongly detected in the animal cancers assessed, pan-deimination detection as assessed by F95 reactivity was overall lower, and detection levels of the five PAD isozymes varied between cancer types, with PAD2 and PAD3 being the most prominent isozymes across the specimens assessed. While explorative, this study provides some new insights into PADs in tumours across diverse vertebrate species and possibly PAD-mediated roles in cancer evolution.

## Figures and Tables

**Table 2:** Overview of the 20 different cancer samples assessed from 12 different animal species in the current pilot study. Animal species name, cancer type, and further information on the samples are listed.

Animal *(Species Name)*	Carcinoma Type	Sample Type
Rabbit *(Oryctolagus cuniculus)*	Mammary adenocarcinoma	Biopsy from the mammary gland of a four-year old female rabbit/doe.
Dog *(Canis lupus familiaris)*	(1) Mixed germ cell-sex cord stromal tumour, testicle; (2) Squamous cell tumour; origin of tissue unknown	Biopsies from seven-year old dog, male: (1) mixed tumour in testicle (based on morphological assessment); (2) Bladder.
Regent parrot *(Polytelis anthopeplus)*	Lymphoma, liver and spleen	Cadaver, male bird. Tumour in various organs, including liver, spleen, heart, kidney, adipose tissue and skin. Diagnosis: disseminated malignant round cell tumour, probable lymphoma.
Domestic duck *(Anas platyrhynchos domesticus)*	Malignant round cell tumour, liver; probably lymphoma	Organ samples. Tumour, liver; probably lymphoma—not verified by IHC; tumour cells only seen in the liver
Mink *(Neovision vision)*	Malignant renal tumour, possibly nephroblastoma	Cadaver, adult mink, male. Tumour in kidney with metastasis to lung and liver.
Cat *(Felis catus)*	Pulmonary carcinoma	Cadaver of 13-year old male cat. Primary lung tumour suspected as no other primary tumour found. Metastasis to heart.
Horse *(Equus caballus)*	Carcinoma in liver (ovary and mesenteric lymph node)	Organs from an 18-year old mare. Carcinoma in liver; probable primary bile duct carcinoma but tumour metastasis to the liver could not be ruled out.
Cattle *(Bos Taurus)*	Neuroendocrine tumour (metastasis to the heart)	Heart with tumour located on the surface of the right ventricle. Origin of the tumour uncertain (can be from chemoreceptors at the carotid or aortic body, from C-cells in the thyroid gland (ultimobranchial tumour or adrenal gland medulla (pheochromocytoma—neuroendocrine tumour).
Dog *(Canis lupus familiaris)*	Anaplastic mammary carcinoma	Biopsy from a five-year old bitch.
Sheep *(Ovis aries)*	Malignant round cell tumour, skin	Two-year old ewe. Skin with tumour was received; origin of this highly malignant tumour is unknown.
Hamster (*Mesocricetus auratus)*	Lymphoma, mesentery/small intestine	Cadaver of a hamster with tumour masses in mesentery. Multicentric lymphoma caused by Hamster Polyomavirus (HaPV).
Dog *(Canis lupus familiaris)*	Scirrhous adenocarcinoma, mammary gland	Biopsy from tumour in mammary gland of an eight-year old bitch
Horse (*Equus caballus)*	Squamous cell carcinoma, penile.	Samples taken from penile tumour.
Cattle *(Bos taurus)*	Metastatic carcinoma, lungs and lymph nodes	Organ samples with tumour in lungs and lymph nodes; origin of the primary tumour unknown
Cat *(Felis catus)*	Malignant tumour, kidneys; probably lymphoma	Kidneys from an eight-month old male cat. Tumour in both kidneys—probably lymphoma.
Dog *(Canis lupus familiaris)*	Granulosa cell tumour, ovary	Samples from ovary of a five-year-old bitch.
Cattle *(Bos Taurus)*	Lymphoma, lymph node	Organ samples from a cow, including kidney and lymph nodes.
Mink *(Neovision vision)*	Malignant renal tumour, probably nephroblastoma	Cadaver of a male mink. Extensive necrosis and haemorrhage in tumour mass.
Reindeer *(Rangifer tarandus)*	Multicentric malignant tumour, probably mesothelioma	Organ samples from reindeer, female. Tumour masses in the abdominal cavity. Spleen and diaphragm with tumour.
Pig *(Sus scrofa domesticus)*	Lymphoma (lymph node)	Organ sample from a slaughtered pig. Lymphoma in lymph node and kidney.

## Data Availability

All data for this study is included within the manuscript.

## Supplementary Materials

The following supporting information can be downloaded at: https://www.mdpi.com/article/10.3390/ijms23158697/s1.
