# Supplementary material for: A Pilot Study on Peptidylarginine Deiminases and Protein Deimination in Animal Cancers across Vertebrate Species
Source: Int J Mol Sci. 2022 Aug 4;23(15):8697. doi: 10.3390/ijms23158697 (PMC9368843; doi:10.3390/ijms23158697)
Supplement: Supplementary file 1 [file ijms-23-08697-s001.zip › ijms-1830689-supplementary.pdf]

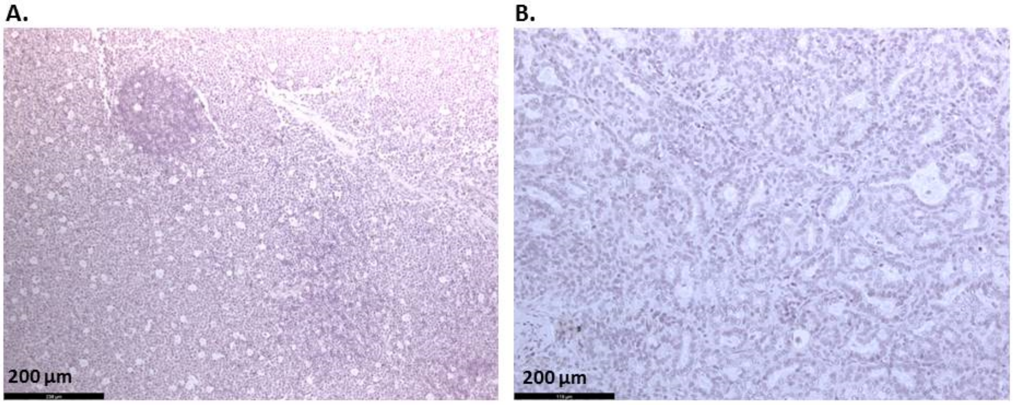

Supplementary Figure S1. Negative control images, showing secondary antibody only controls, omitting the primary antibodies. **A.** Secondary anti-Rabbit IgG antibody (used for all PAD isozyme and CitH3 stainings); **B.** Secondary anti-Mouse IgM antibody (used for F95 stainings); scale bars represent 200 µm.
